# Source-Specific Volatile Organic Compounds and Emergency Hospital Admissions for Cardiorespiratory Diseases †

**DOI:** 10.3390/ijerph17176210

**Published:** 2020-08-27

**Authors:** Jinjun Ran, Marianthi-Anna Kioumourtzoglou, Shengzhi Sun, Lefei Han, Shi Zhao, Wei Zhu, Jinhui Li, Linwei Tian

**Affiliations:** 1 School of Public Health, The University of Hong Kong, Hong Kong, China; jimran@hku.hk (J.R.); szsun@bu.edu (S.S.); linweit@hku.hk (L.T.); 2 Department of Environmental Health Sciences, Columbia University, New York, NY 10027, USA; mk3961@cumc.columbia.edu; 3 Department of Environmental Health, Boston University School of Public Health, Boston, MA 02118, USA; 4 School of Nursing, The Hong Kong Polytechnic University, Hong Kong, China; le-fei.han@connect.polyu.hk; 5 JC School of Public Health and Primary Care, Chinese University of Hong Kong, Hong Kong, China; zhaoshi.cmsa@gmail.com; 6 Department of Toxicology, Guangzhou Center for Disease Control and Prevention, Guangzhou 510440, China

**Keywords:** volatile organic compounds, source apportionment, cardiovascular disease, respiratory disease, emergency hospital admissions

## Abstract

Knowledge gaps remain regarding the cardiorespiratory impacts of ambient volatile organic compounds (VOCs) for the general population. This study identified contributing sources to ambient VOCs and estimated the short-term effects of VOC apportioned sources on daily emergency hospital admissions for cardiorespiratory diseases in Hong Kong from 2011 to 2014. We estimated VOC source contributions using fourteen organic chemicals by positive matrix factorization. Then, we examined the associations between the short-term exposure to VOC apportioned sources and emergency hospital admissions for cause-specific cardiorespiratory diseases using generalized additive models with polynomial distributed lag models while controlling for meteorological and co-pollutant confounders. We identified six VOC sources: gasoline emissions, liquefied petroleum gas (LPG) usage, aged VOCs, architectural paints, household products, and biogenic emissions. We found that increased emergency hospital admissions for chronic obstructive pulmonary disease were positively linked to ambient VOCs from gasoline emissions (excess risk (ER%): 2.1%; 95% CI: 0.9% to 3.4%), architectural paints (ER%: 1.5%; 95% CI: 0.2% to 2.9%), and household products (ER%: 1.5%; 95% CI: 0.2% to 2.8%), but negatively associated with biogenic VOCs (ER%: −6.6%; 95% CI: −10.4% to −2.5%). Increased congestive heart failure admissions were positively related to VOCs from architectural paints and household products in cold seasons. This study suggested that source-specific VOCs might trigger the exacerbation of cardiorespiratory diseases.

## 1. Introduction

Volatile organic compounds (VOCs) are organic chemicals which can be exhausted or evaporated into the air under normal atmospheric conditions of temperature and pressure from combustion or non-combustion sources. Emissions of VOCs are regulated in many cities to prevent the formation of ozone (O_3_) and secondary organic aerosol (SOA) which contribute to the photochemical pollution [1,2]. Numerous studies further observed the direct linkage of VOCs with cardiorespiratory diseases, but mostly in occupational settings or animal models [3,4,5,6,7,8,9].

Specifically, exposure to indoor VOCs may result in chronic bronchitis, an allergic reaction and diminution of pulmonary function which are typical symptoms of chronic obstructive pulmonary disease (COPD) and asthma [3,4,5,6,7,10,11]. The potential pathogenesis of VOC’s respiratory toxicity includes inflammation, oxidative stress, and the dysfunction of pulmonary surfactant [6,12,13]. Personal exposure to specific VOCs, such as benzene and toluene, were also found to be linked with cardiovascular dysfunctions, probably because of the systemic inflammation and arrhythmia [8,9,14,15,16]. However, these studies mainly focused on the long-term cardiorespiratory effects of indoor VOC chemicals. Evidence is still limited on the association between short-term exposure to ambient VOCs and cardiorespiratory diseases in the general population [17,18,19]. Moreover, VOC-induced effects may be specific to the original location since the VOC chemicals emitted from the same source may have similar physicochemical composition and toxicity [20,21,22]. To our knowledge, there is no study exploring the potential short-term effects of source-apportioned VOCs. Therefore, we took advantage of ambient VOC speciation data to identify their apportioned sources by positive matrix factorization (PMF), then to estimate the short-term associations between source-apportioned VOCs and emergency hospital admissions for both cardiovascular and respiratory diseases in the Hong Kong population by a time-series study.

## 2. Materials and Methods

### 2.1. Outcome Assessment

Daily counts of emergency hospital admissions between 1 April 2011 and 31 December 2014 were collected from the Hospital Authority which is a statutory body responsible for forty-two Hong Kong public hospitals, covering approximately 90% of hospital beds in Hong Kong for local residents [23]. The principal diagnosis on discharge was recorded according to the International Classification of Disease, ninth version (ICD-9). Hospital admissions for all-cause cardiovascular disease (CVD; ICD-9: 390–459), ischemic heart disease (IHD; ICD-9: 410–414), congestive heart failure (CHF; ICD-9: 428), all-cause respiratory disease (RD; ICD-9: 460–519), chronic obstructive pulmonary disease (COPD; ICD-9: 491, 492 and 496), and asthma (ICD-9: 493) were extracted and used in our analyses. Other information, such as sex, year of birth and date of admission, was available in the records. Time series by age subgroup (age < 65 and age ≥ 65 years) and sex (female and male) subgroup were constructed for stratification analysis.

### 2.2. Exposure and Covariate Assessment

Daily concentrations of VOC species were obtained from the Environment Protection Department of Hong Kong (HKEPD), a government-run ambient monitoring network. The real-time VOCs’ concentrations were monitored in four stations, located in Mong Kok (MK), Tung Chung (TC), Yuen Long (YL) and the Hong Kong University of Science and Technology (UT), covering urban (MK), suburban (TC and YL), and rural (UT) areas (Figure S1). Details about the collection, analysis and calibration processes have been previously described [24,25,26,27]. After removing the species with more than 35% missing values or below detection limits (BDL), a total of 14 species remained and were used for the source apportionment analysis: ethane, propane, iso-butane, n-butane, iso-pentane, n-pentane, propene, isoprene, hexane, benzene, toluene, ethylbenzene, m/p-xylene and o-xylene [24,25]. In general, the detection limits of the included species ranged from 2 to 56 ppt. The accuracy and precision of the measurements ranged between 1–7% and 1–10%, respectively. Because the outcome in our analysis was the daily counts of emergency cardiorespiratory hospital admissions across the whole city, the corresponding exposure should also be the territory-wide daily mean concentrations of pollutants. We thus estimated the territory-wide mean concentrations of the fourteen VOC chemicals by averaging the concentrations from the four monitoring stations. Daily mean concentrations of relevant trace gases (CO and O_3_) across ten general fixed-site monitoring stations were also obtained from HKEPD. Daily weather conditions in the same period, including information on temperature and relative humidity, were provided by the Hong Kong Observatory.

### 2.3. Statistical Analysis

We used positive matrix factorization (PMF) version 5.0 (Environmental Protection Agency, the United States. Available online: https://www.epa.gov/air-research/positive-matrix-factorization-model-environmental-data-analyses) for the receptor-based source apportionment of VOC species. PMF is a multivariate factor analysis tool that is widely used for particulate matter, toxic air, aerosol, and VOC data. The PMF procedure and relevant mathematical equations were detailed in our Supplementary Materials, and its relevant application in VOC species has also been frequently illustrated elsewhere [25,27,28,29,30,31]. Briefly, the concentration file was input into the PMF model after the unit of each VOC species was converted from mixed ratios (ppt) into mass concentrations (µg/m^3^). Daily mean CO was involved as the tracer of incomplete combustion [25]. The equation-based uncertainty file was used to provide species-specific parameters to calculate uncertainties for each species. Q_true_ and Q_robust_ are fundamental and critical parameters which are used to assess the goodness-of-fit between observations and predictions in PMF. Twenty base runs were conducted, allowing for the evaluation of the variation in Q, and the run with the lowest Q value was selected as the solution. Four to eight factors with various values of F_peak_ were tested considering previous papers about VOC source apportionment in Hong Kong [25,27,30]. Q_robust_ values, G-space plots and residual distributions were used to filter out the optimal solution with the most realistic rotation. Eventually, six factors were identified in this study.

We applied quasi-Poisson models to estimate the association between VOC sources and emergency hospital admissions for cardiorespiratory diseases. We selected a priori the degrees of freedom (*df*) for the time-dependent variables, based on previous studies in the area. Specifically, we used natural cubic splines with eight *df* per year for long-term and seasonal trends, six *df* for the daily mean temperature at lag 0, as well as at average lag 1–3, and three *df* for the daily mean relative humidity at lag 0, as well as at average lag 1–3 [20]. Public holidays and day-of-week (DOW) were included as indicator variables in our model. We applied distributed-lag models (DLM) to pre-build the exposure–lag–response relation for each factor of VOC sources [32]. We used the second-degree polynomial for the lag constraint to allow the effect estimates to vary smoothly in time. We also adjusted for ozone using distributed lag terms with the same parameterization as illustrated above for the VOC sources [33]. We assessed the model fit using residual and partial autocorrelation function (PACF) figures. When autocorrelation was observed in the first two lags, we additionally adjusted for autoregressive terms for the outcome variable in the core model to reduce autocorrelation [34].

Stratification analyses for sex (female and male) and age (<65 and ≥65) were conducted to further check the susceptible subpopulations. Sensitivity analyses were also performed to test the robustness of the associations using the following strategies: (1) controlling for temperature lagged effect extended to one and two weeks; (2) controlling for the cumulative-lag effect of ozone extended to one and two weeks; (3) modifying the *df* for time trends from eight to four and twelve. We presented the effect estimates as the percent change in the excess risk (ER%) of cause-specific admission per interquartile range (IQR) increase in the concentrations of each source: ER% = (RR − 1) × 100%. All analyses were performed in environment R 3.6.1 version.

## 3. Results

**Figure 1:**
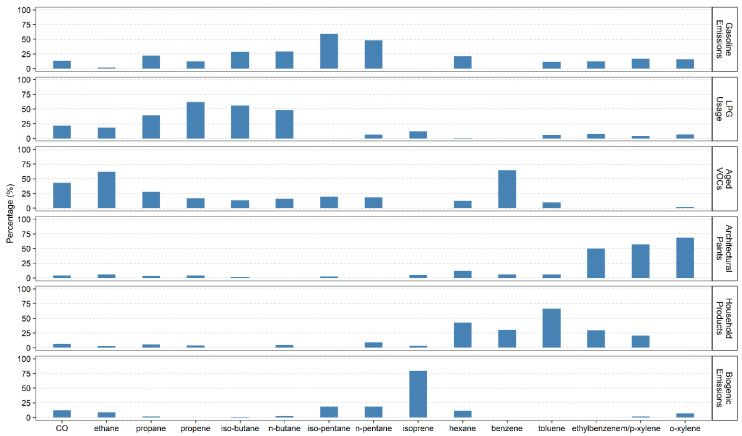
Explained variations of species (% of species sum) in six identified sources in Hong Kong, April 2011 to December 2014, estimated by positive matrix factorization (PMF). LPG, liquefied petroleum gas; VOC, volatile organic compounds.

A solution with six factors presented the most feasible results, namely gasoline emissions (both exhaust and evaporation), liquefied petroleum gas (LPG) usage, aged VOCs, architectural paints, household products, and biogenic emissions. Figure 1 shows the resolved source profiles (% of species sum), and the identification process was consistent with previous studies in Hong Kong [25,26,27,30]. From April 2011 to December 2014, LPG usage took the largest fraction (30%) of the collected total VOCs, followed by gasoline emissions (22%) and aged VOCs (20%). VOCs from architectural paints and household products accounted for about 22% in total, and biogenic VOCs accounted for the smallest faction (6%). We identified the factor as aged VOCs because the factor was mainly composed of less reactive species, such as the ethane and benzene as well as the ratio of benzene and toluene (B/T ratio) was about 1.7 in the factor. The B/T ratio is an indicator on the age of the air masses, and the ratio is much higher in aged air than that in the fresh air. The B/T ratio was about 1.7 in our derived profile, much higher than the typical value from fresh vehicle exhaust (~0.5) and non-combustion source (even lower) based on previous studies [30,35]. In the study period, daily mean emergency hospital admissions for all-cause cardiovascular and respiratory diseases were about 202 and 285, respectively, including 37 for IHD, 39 for CHF, 55 for COPD, and 18 for asthma. The daily mean temperature was 23.8 °C and relative humidity was 78.7% (Table 1).

Seasonal and secular patterns for VOC sources are illustrated in Figure S2. Concentrations of aged VOCs, architectural paints and household products remained higher in winter and lower in summer. However, the ambient concentration of biogenic VOCs followed a reversed seasonality. The long-term trend of VOCs from LPG usage is slightly declining from 2013 to 2014. Pearson correlation coefficients across source-specific contributions are shown in Table 2. Two-by-two correlations between sources were generally nil to moderate. Two exceptions were the correlation between architectural paints and household products (*r* = 0.72), and the correlation between biogenic VOCs and aged VOCs (*r* = −0.70).

**Figure 2:**
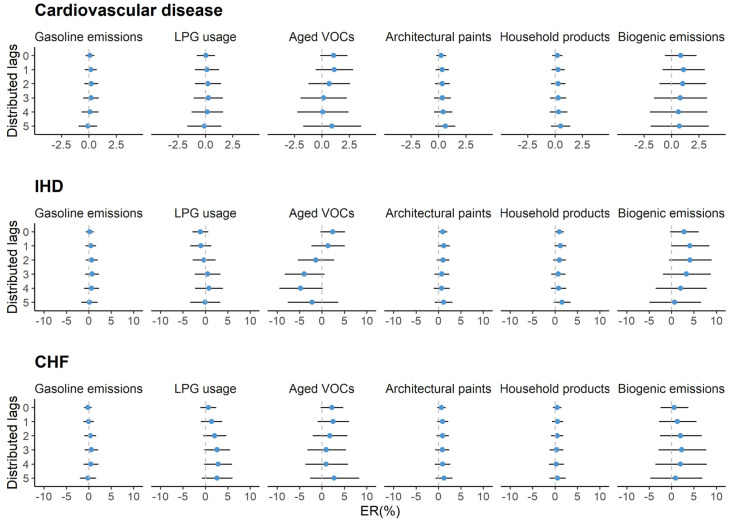
Percent excess risk (ER%) with the corresponding 95% confidence interval (95% CI) in emergency hospital admissions for cause-specific cardiovascular diseases per IQR increment in VOC sources at different distributed-lag periods in Hong Kong from April 2011 to December 2014. IHD, ischemic heart disease; CHF, congestive heart failure. The red point indicates the estimate of statistical significance (*p*-value < 0.05) and the blue point indicates the non-significant estimate (*p*-value ≥ 0.05).

**Figure 3:**
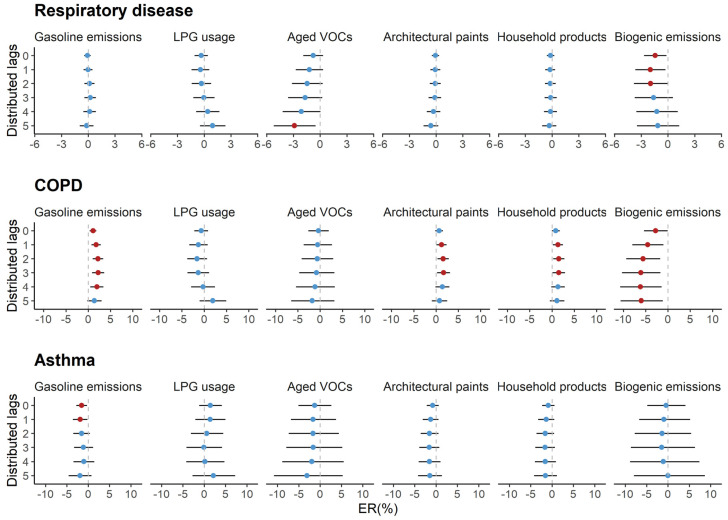
Percent excess risk (ER%) with the corresponding 95% confidence interval (95% CI) in emergency hospital admissions for cause-specific respiratory diseases per IQR increment in VOC sources at different distributed-lag periods in Hong Kong from April 2011 to December 2014. COPD, chronic obstructive pulmonary disease. The red point indicates the estimate of statistical significance (*p*-value < 0.05) and the blue point indicates the non-significant estimate (*p*-value ≥ 0.05).

In all seasons, we did not observe clear associations of emergency hospital admissions for cause-specific cardiovascular diseases with short-term exposure to VOC apportioned sources (Figure 2). However, we found that an increased risk of emergency COPD hospital admissions was linked to an IQR increase in VOCs from gasoline emissions (2.1%; 95% CI: 0.9%, 3.4%), architectural paints (1.5%; 95% CI: 0.2%, 2.9%), and household products (1.5%; 95% CI: 0.2%, 2.8%) at lag 0–2. Biogenic VOCs were negatively associated with the emergency COPD hospital admissions and the estimated ER% was −6.6% (95% CI: −10.4%, −2.5%) (Figure 3). In cold seasons, an IQR increment in VOCs from architectural paints and household products were positively related to the increase in emergency CHF hospital admissions and their estimated ER%s were 4.1% (95% CI: 1.2%, 7.1%) and 3.5% (95% CI: 0.8%, 6.2%), respectively (Table 3). However, the seasonal variation was not evident in the associations with respiratory hospital admissions (Table 4). Results remained stable when modifying the lags of temperature and ozone control in our models. Fluctuation was observed when resetting the degrees of freedom for time trends, especially when four degrees of freedom were used in our models (Figure S3). We observed little evidence supporting the effect modifications of age and sex on the associations (Table S1).

## 4. Discussion

To the best of our knowledge, this is the first study directly estimating the health effects of source-apportioned VOCs in the Hong Kong population. We identified a six-source solution for collected ambient VOCs, namely gasoline emissions, LPG usage, aged VOCs, architectural paints, household products, and biogenic emissions. The identification process for source categories were referred to previous studies in Hong Kong [25,26,30]. However, the source solutions were not consistent since a different number of monitoring stations was adopted in these studies. Specifically, the studies by Ou et al. and Guo et al. collected the VOC data from only one station (Tung Chung) set in a suburban area [25,26]. Lau at al. averaged the VOC data from four sites to represent the city-wide mean concentration including one roadside monitoring station in a urban area, two stations in suburban areas and one in a rural area, which is similar with our study [30].

Higher concentrations of biological VOCs were seen in summer when compared to those in winter (Figure S2). This was probably because the photosynthesis rate elevates exponentially with temperature [36]. In contrast, the contribution of aged VOCs, which mainly contain relatively unreactive components, such as ethane and benzene, shows an inverse seasonal pattern probably because of the following three reasons. First, the prevailing wind in summer is south and southwest. Unreactive VOCs from the mainland cannot migrate to Hong Kong in summer because Hong Kong is in the south of China. Second, the invasion of the cold front from the northeast and northwest in winter leads to the deceleration of air motion in Hong Kong, which can result in insufficient mixing and the elimination of local aged VOCs. The seasonality of aged VOCs were in line with the findings in the study of Lau et al. [30]. It is noteworthy that we identified the factor as “aged VOCs” based on its dominated VOC chemicals and B/T ratio in our and Lau’s studies [30]. The aged VOCs here did not refer to the oxidation products of VOCs. Their oxidation products may have a different seasonal pattern and result in higher risk in summertime. For other sources in common daily use, such as architectural paints and household products, we can detect an inconspicuous and similar seasonal pattern to aged VOCs. From the source-related correlation coefficients, the positive correlation between architectural paints and household products was observed probably because the VOC mixture was mainly released from residential homes, including evaporating or volatilizing from paints, solvents, or cleaning products. The negative correlation between aged VOCs and biogenic VOCs could be related to their seasonality and chemical reactivity in the atmosphere. Isoprene is highly reactive in the atmosphere and the aged VOC factor is composed of less reactive species. The higher outdoor temperature and intense sunlight in summer increase the emission of isoprene. However, in winter, aged VOCs could accumulate more because of their lesser reactivity.

In our study, we found positive associations of VOCs from gasoline emissions, architectural paints, and household products with an increased risk of emergency COPD hospital admissions. The effects of gasoline emissions on the respiratory system were suggested to be related to the inflammation and oxidant stress based on multiple rodent biological models [37]. Since few studies indicated the respiratory effects of pentane, the adverse effects of gasoline emissions could be dominated by toluene, ethylbenzene, and xylene (TEX), as well as other gaseous pollutants from gasoline exhausts, such as nitrogen oxides. Likewise, TEX accounts for the majority in VOCs from architectural paints and household products. The detected positive associations with COPD were in line with previous findings who illustrated the potential effects of TEX on the diminution of lung function and airway inflammation [5,6,38]. Yoon et al. observed that urinary levels hippuric acid and methyl-hippuric acid (biomarkers for toluene and xylene, respectively) were related to the drop of forced expiratory volume in 1 s (FEV_1_) and FEV1/forced vital capacity (FVC). They further found that exposure to TEX was associated with markers of oxidative stress, suggesting that oxidative stress could be involved in the pathogenesis of the loss of lung function associated with TEX [6]. Another study conducted by Martin et al. also found the significant associations between the exposure to toluene and ethylbenzene and a reduction of FEV1 and the elevated acidity of exhaled breath condensate, suggesting that the exposure to TEX could result in airway changes and inflammation in children [5]. Atopy, a typical category of allergy, was also illustrated to be a risk factor for acute respiratory symptoms in COPD patients, probably because serum Immunoglobulin E (IgE) might accelerate the decline of lung function [39,40]. However, it is still uncertain if ambient concentrations of gasoline-related VOCs can trigger the allergic response since our results did not observed a significant association between gasoline-related VOCs and asthma hospitalizations, which were more relevant to allergic response. Notably, an emerging study found that the inhaled TEX were concerned with surface tension, phase behavior and the solubilization of pulmonary surfactant then might generate acute or chronic effects on respiratory functions, such as COPD exacerbation [12].

VOCs from biogenic emissions were observed to be negatively related to COPD hospitalizations. Considering that isoprene accounts for 80% of total biogenic VOCs, the negative effect could be associated with ambient isoprene. Isoprene is a decomposed compound from terpene which is produced from the chloroplasts of many plants, and is also a major hydrocarbon found in human breath. Breath isoprene is proposed as a noninvasive marker of physiological response to oxidative stress to epithelial membranes and is elevated along with the inflammation status [41,42]. However, the role of isoprene in the human body is still unclear because of the deficient understanding in its biologic function. In our study, we found that biogenic VOCs were negatively associated with emergency COPD hospital admissions. The biological VOCs were considered as a surrogate factor of green spaces because the protective effects of green spaces on COPD were widely documented [43,44,45]. However, animal studies suggested that isoprene oxidation products might contribute to the formation of secondary organic aerosol, an enhanced sensory irritant, and airflow limitation responses [46]. More epidemiological and animal studies are warranted to make the paradox clear.

VOCs from architectural paints and household products were shown to be associated with emergency CHF hospital admissions in cold seasons. Biphasic tachycardia and arrhythmia might be involved in the biomechanism from non-combustion VOCs and CHF [16,47]. Mentioned above, the dominated constituents are hexane and TEX in VOCs from architectural paints and household products. An animal study suggested that oral toluene exposure could result in biphasic tachycardia, and then lead to a rise in blood pressure [16]. Ma et al. also observed that occupational exposure to VOCs in a hair salon could lead to increases in serum C-reactive protein and 8-hydroxy-2′-deoxyguanosine, as well as falls in indices of heart rate variability [47]. However, the cardiovascular effects of VOCs from architectural paints and household products were significantly observed in cold seasons rather than warm seasons. Previous studies in Hong Kong illustrated that moderate cold weather was individually responsible for a considerable attributable risk for cardiovascular diseases and could also enhance the adverse effects of air pollution on emergency cardiovascular functions [48,49]. Moreover, organic constituents from architectural paints and household products have higher concentrations in cold seasons than those in warm seasons.

Our findings should be interpreted with caution. First, VOCs from architectural paints and household products accounted for about 21% of the collected total VOCs, which could be lower than their real concentrations in residential exposure [50]. One explanation is that one roadside monitoring station (MK) collected more VOCs from LPG and gasoline exhausts, thus the VOCs from other sources were accordingly underestimated at the average level. Second, to ensure the quality of source apportionment, numerous volatile organic constituents with over 40% missing values, were excluded before PMF analysis, such as alkene, alkyne, aldehyde, and ketone. Therefore, we cannot estimate the health effects of these compounds to compare with the findings of an emerging study [17]. Third, exposure misclassification error might exist because VOC concentrations are spatially heterogeneous within a city. The territory-wide VOC concentrations in the study were averaged from four monitoring stations, which might reduce the variations of each organic chemical. Our estimates hence tend to underestimate the true health effects of ambient VOCs [33]. Further studies would be extended to minimize the limitation by performing the PMF on the individual station data and using hourly VOC data resolution as well as utilizing the data of wind direction to help identify derived sources at each measurement site. Fourth, effect modifications by other potential factors, such as body mass index or resident location, were not extended in our analysis because we did not have the aggregated information of these factors. Additionally, emergency hospital admissions were aggregated based on primary discharge diagnosis. It inevitably missed numerous patients who did not have severe clinical signs and symptoms or who were served in private sectors, which might underestimate their health impacts likewise [19].

## 5. Conclusions

In summary, we found positive associations of VOCs from gasoline emissions, architectural paints and household products and a negative association of biogenic VOCs with emergency COPD hospital admissions. We also observed positive associations of VOCs from architectural paints and household products with emergency CHF hospitalizations in cold seasons. This study suggests that ambient VOCs might trigger the exacerbation of both cardiovascular and respiratory diseases, especially CHF and COPD. However, only a time-series study conducted in one city is not enough to estimate the potential impacts of ambient VOC exposure on human cardiorespiratory systems. More studies are warranted to further evidence the health effects of ambient VOCs, including short-term exposure in other populations or cities, long-term exposure by cohort studies, and potential toxicological mechanisms by cells or animal experiments.

## Figures and Tables

**Table 1:** Descriptive statistics for daily emergency hospital admissions for cause-specific cardiorespiratory diseases, source-apportioned VOCs, weather conditions and trace gases in Hong Kong from April 2011 to December 2014.

	Mean	SD	25th	50th	75th	IQR
**Emergency hospital admissions (counts)**						
CVD	202	28	182	199	218	36
IHD	37	8	31	36	41	10
CHF	39	12	31	37	46	15
RD	285	60	240	271	322	82
COPD	55	14	44	53	64	20
Asthma	18	6	14	18	22	8
**Source-apportioned VOC (ug/m^3^)**						
Gasoline emissions	8.9	6.2	5.1	7.5	11.2	6.1
LPG usage	11.9	5.6	7.7	11.9	15.5	7.7
Aged VOC	8.1	6.1	2.8	7.3	12.0	9.1
Architectural paints	3.5	3.9	1.0	2.0	4.6	3.6
Household products	5.2	5.7	1.6	3.0	7.1	5.5
Biogenic emissions	2.4	1.8	0.8	2.1	3.7	2.9
**Meteorological conditions**						
Temperature (°C)	23.8	5.2	19.8	25.1	28.3	8.5
Relative humidity (%)	78.7	10.4	74.0	79.0	86.0	12.0
**Trace gases**						
CO (mg/m^3^)	0.9	0.3	0.7	0.9	1.0	0.4
O_3_ (ug/m^3^)	35.1	21.8	19.2	28.5	46.8	27.6

Abbreviations: VOCs, volatile organic compounds; LPG, liquefied petroleum gas CVD, cardiovascular disease; IHD, ischemic heart disease; CHF; congestive heart failure; RD, respiratory disease; COPD, chronic obstructive pulmonary disease; IQR, interquartile range; SD, standard deviation; CO, carbon monoxide; O_3_, ozone.

**Table 2:** Spearman correlation coefficients between the estimated concentrations of VOC sources in Hong Kong from April 2011 to December 2014.

	Gasoline Emissions	LPG Usage	Aged VOCs	Architectural Paints	Household Products	Biogenic Emissions
Gasoline emissions	1.00					
LPG Usage	0	1.00				
Aged VOCs	0.19	−0.28	1.00			
Architectural paints	0.38	−0.20	0.42	1.00		
Household products	0.45	−0.20	0.50	0.72	1.00	
Biogenic emissions	−0.27	−0.02	−0.70	−0.34	−0.41	1.00

Abbreviations: VOC, volatile organic compounds; LPG, liquefied petroleum gas.

**Table 3:** Percent excess risks (%) of emergency hospital admissions for cardiovascular diseases with VOC sources at distributed 0–2 lags across seasons in Hong Kong from 2011 to 2014 ^a^.

	All Seasons	Seasonal Variation		
		Cold Seasons	Warm Seasons	*p*-Value ^b^
Cardiovascular disease				
Gasoline emissions	0.1 (−0.6, 0.8)	0 (−1.1, 1.1)	0.1 (−0.8, 1.0)	0.902
LPG usage	0 (−1.3, 1.3)	0.5 (−1.3, 2.4)	−0.6 (−2.3, 1.1)	0.385
Aged VOCs	0.5 (−1.5, 2.5)	0.5 (−1.3, 2.2)	−0.4 (−2.6, 1.9)	0.571
Architecture paints	0.3 (−0.4, 1.0)	0.6 (−0.8, 2.0)	0.4 (−0.5, 1.3)	0.851
Household products	0.3 (−0.4, 1.0)	0.6 (−0.7, 1.9)	0.3 (−0.3, 0.9)	0.740
Biogenic emissions	1.0 (−1.3, 3.3)	0.9 (−0.9, 2.7)	1.2 (−0.8, 3.4)	0.799
IHD				
Gasoline emissions	0.6 (−0.8, 2.1)	0 (−2.2, 2.3)	0.7 (−1.4, 2.8)	0.651
LPG usage	0.2 (−2.5, 2.9)	0.9 (−3.3, 5.4)	−0.1 (−3.4, 3.3)	0.705
Aged VOCs	−2.5 (−6.7, 1.8)	−1.1 (−4.6, 2.6)	−5.6 (−10.3, −0.6)	0.148
Architecture paints	0.9 (−0.6, 2.5)	1.2 (−1.6, 4.1)	2.0 (0.1, 4.0)	0.636
Household products	1.0 (−0.5, 2.5)	1.8 (−0.8, 4.4)	1.1 (−0.3, 2.5)	0.662
Biogenic emissions	2.9 (−1.9, 8.0)	2.1 (−1.5, 5.8)	2.3 (−2.4, 7.3)	0.935
CHF				
Gasoline emissions	0.3 (−1.1, 1.7)	1.1 (−1.2, 3.5)	−1.0 (−2.9, 1.0)	0.180
LPG usage	2.3 (−0.4, 5.0)	3.6 (−0.2, 7.7)	1.3 (−2.5, 5.4)	0.422
Aged VOCs	0.3 (−3.7, 4.4)	0.4 (−3.2, 4.1)	−0.7 (−5.5, 4.4)	0.733
Architecture paints	0.7 (−0.7, 2.2)	4.1 (1.2, 7.1)	−1.4 (−3.3, 0.5)	0.002
Household products	0.4 (−1.0, 1.9)	3.5 (0.8, 6.2)	−0.7 (−2.1, 0.6)	0.006
Biogenic emissions	2.6 (−2.2, 7.7)	1.9 (−1.8, 5.7)	3.2 (−1.4, 8.1)	0.669

Abbreviations: VOC, volatile organic compounds; LPG, liquefied petroleum gas; IHD, ischemic heart disease; CHF, congestive heart failure; ^a^ distributed-lag model over the previous three days (lag_0–2_).^b^ Wald test, to test the significance of difference across seasons.

**Table 4:** Percent excess risks (%) of emergency hospital admissions for respiratory diseases with VOC sources at distributed 0–2 lags across seasons in Hong Kong from 2011 to 2014 ^a^.

	All Seasons	Seasonal Variation		
		Cold Seasons	Warm Seasons	*p*-Value ^b^
Respiratory disease				
Gasoline emissions	0.3 (−0.3, 0.9)	0 (−1.0, 0.9)	0.3 (−0.5, 1.2)	0.567
LPG usage	−0.1 (−1.2, 1.1)	−0.4 (−1.9, 1.2)	1.1 (−0.5, 2.8)	0.211
Aged VOCs	−1.4 (−3.2, 0.5)	−1.1 (−2.7, 0.5)	−1.2 (−3.3, 0.9)	0.934
Architecture paints	−0.2 (−0.8, 0.5)	−0.2 (−1.4, 1.0)	0.6 (−0.2, 1.4)	0.293
Household products	−0.1 (−0.7, 0.6)	−0.1 (−1.2, 1.0)	0.3 (−0.3, 0.9)	0.573
Biogenic emissions	−2.2 (−4.1, −0.2)	−1.2 (−2.6, 0.3)	−0.8 (−2.7, 1.1)	0.769
COPD				
Gasoline emissions	2.1 (0.9, 3.4)	0.2 (−1.7, 2.1)	2.9 (1.1, 4.8)	0.048
LPG usage	−0.7 (−3.0, 1.7)	−0.2 (−3.3, 3.1)	−0.8 (−4.3, 2.8)	0.784
Aged VOCs	−1.8 (−5.4, 2.0)	−1.6 (−4.6, 1.6)	−2.2 (−6.6, 2.3)	0.806
Architecture paints	1.5 (0.2, 2.9)	−0.2 (−2.6, 2.3)	3.1 (1.3, 4.9)	0.035
Household products	1.5 (0.2, 2.8)	0.5 (−1.7, 2.7)	1.6 (0.4, 2.8)	0.400
Biogenic emissions	−6.6 (−10.4, −2.5)	0.1 (−2.9, 3.2)	−5.3 (−9.2, −1.2)	0.039
Asthma				
Gasoline emissions	−1.4 (−3.4, 0.7)	−4.1 (−7.3, −0.8)	−1.0 (−4.0, 2.1)	0.165
LPG usage	0.8 (−3.2, 4.8)	−2.9 (−8.1, 2.5)	5.6 (−0.6, 12.1)	0.041
Aged VOCs	−1.3 (−7.4, 5.1)	−0.3 (−5.4, 5.2)	−0.6 (−7.9, 7.2)	0.935
Architecture paints	−1.3 (−3.5, 0.9)	−0.4 (−4.4, 3.8)	−0.4 (−3.3, 2.5)	0.980
Household products	−1.4 (−3.5, 0.8)	−0.9 (−4.6, 2.9)	−0.3 (−2.4, 1.8)	0.768
Biogenic emissions	−2.3 (−9.0, 4.8)	−1.4 (−6.5, 3.9)	0.3 (−6.5, 7.6)	0.696

Abbreviations: VOC, volatile organic compounds; LPG, liquefied petroleum gas; COPD, chronic obstructive pulmonary disease. ^a^ distributed-lag model over the previous three days (lag_0–2_). ^b^ Wald test, to test the significance of difference across seasons.

## Supplementary Materials

The following are available online at https://www.mdpi.com/1660-4601/17/17/6210/s1, Figure S1: Distribution of the four VOC monitoring stations in Hong Kong; Figure S2: Time series plots of the daily mean concentrations of VOC sources in Hong Kong, 2011-2014, Figure S3: Sensitivity analyses for the associations of VOC sources with emergency hospital admissions for total and cause-specific diseases in Hong Kong, 2011-2014; Table S1: Percent excess risks (%) of emergency hospital admissions for cardiovascular and respiratory diseases with VOC sources at distributed 0-2 lags across seasons in Hong Kong from 2011 to 2014.
