# Supplementary material for: Source-Specific Volatile Organic Compounds and Emergency Hospital Admissions for Cardiorespiratory Diseases
Source: Int J Environ Res Public Health. 2020 Aug 27;17(17):6210. doi: 10.3390/ijerph17176210 (PMC7503811; doi:10.3390/ijerph17176210)
Supplement: Supplementary file 1 [file ijerph-17-06210-s001.pdf]

Title: **Source-specific Volatile Organic Compounds and Emergency Hospital Admissions for Cardiorespiratory Diseases**

Supplementary Material

1. PMF procedure.. 2
2. Distribution of the four VOC monitoring stations in Hong Kong 3
3. Time series plots of the daily mean concentrations of VOC apportioned sources 4
4. Sensitivity analyses for the associations 5
5. Age and sex stratified analyses 6

1. PMF procedure

PMF generated a set of source profile by solving a weighted and constrained least-squares optimization equation. The mathematical mass balance equation is written as [1]:

$$x_{ij} = \sum_{k=1}^{p} g_{ik} f_{kj} + e_{ij}$$

where g_{ik} indicates the source concentration from the k_{th} (k = 1, 2, …, p sources) to the i_{th} sample; f_{kj} stands for the element fraction of j_{th} in the k_{th}; and e_{ij} is for the residual for each observation.

The objective function Q(E) was generated based on the inherent uncertainties for each observation, which is defined as:

$$\mathrm{Q(E)} = \sum_{j=1}^{m} \sum_{j=1}^{n} \left[\frac{e_{ij}}{s_{ij}}\right]^2$$

where s_{ij} represents the estimate for uncertainty from the j_{th} species in the i_{th} date.

The uncertainty is calculated by $\sqrt{(EF \times conc)^2 + (MDL)^2}$ when the concentration is more than MDL, where EF means an error fraction (EF = the percent uncertainty × 100), and MDL is the method detection limit. And the uncertainty is estimated by 5/6 MDL when the concentration is less than or equal to MDL [2].

2. Distribution of the four VOC monitoring stations in Hong Kong

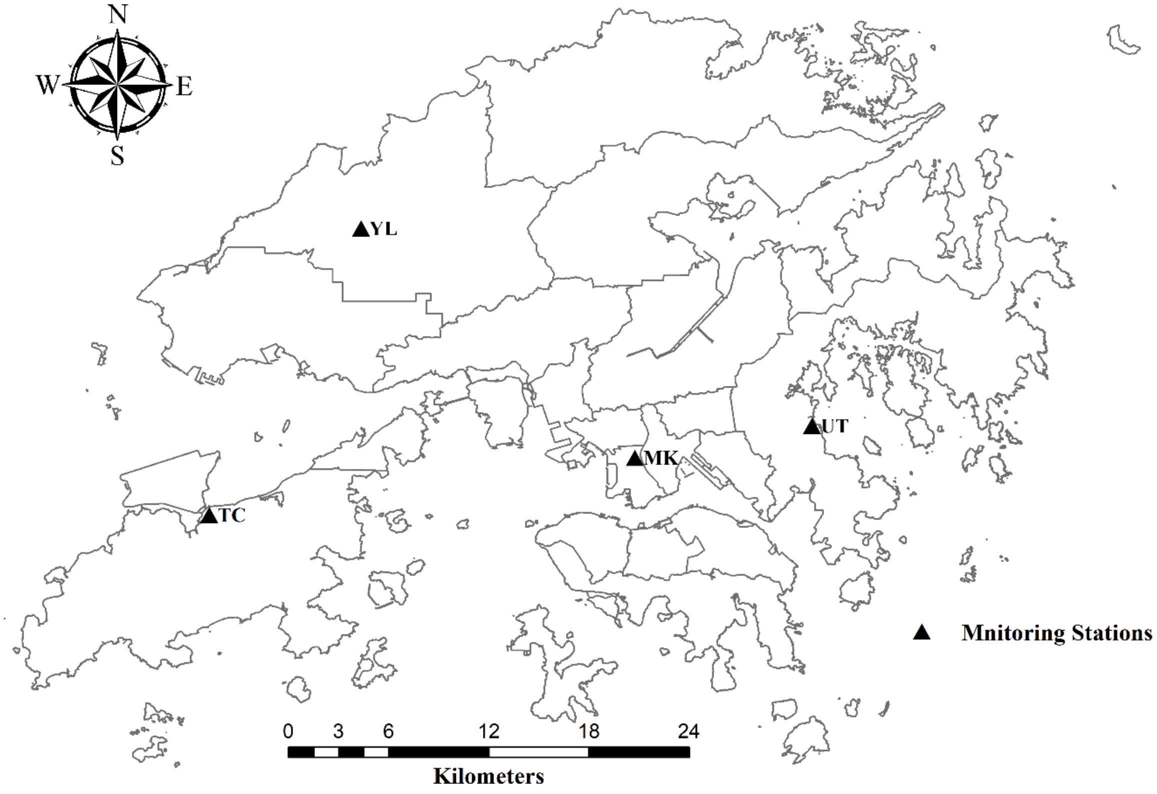

Fig. S1: Distribution of the four VOC monitoring stations in Hong Kong. MK, Mong Kok; TC, Tung Chung; YL, Yuen Long; UT, the Hong Kong University of Science and Technology.

3. Time series plots of the daily mean concentrations of VOC apportioned sources

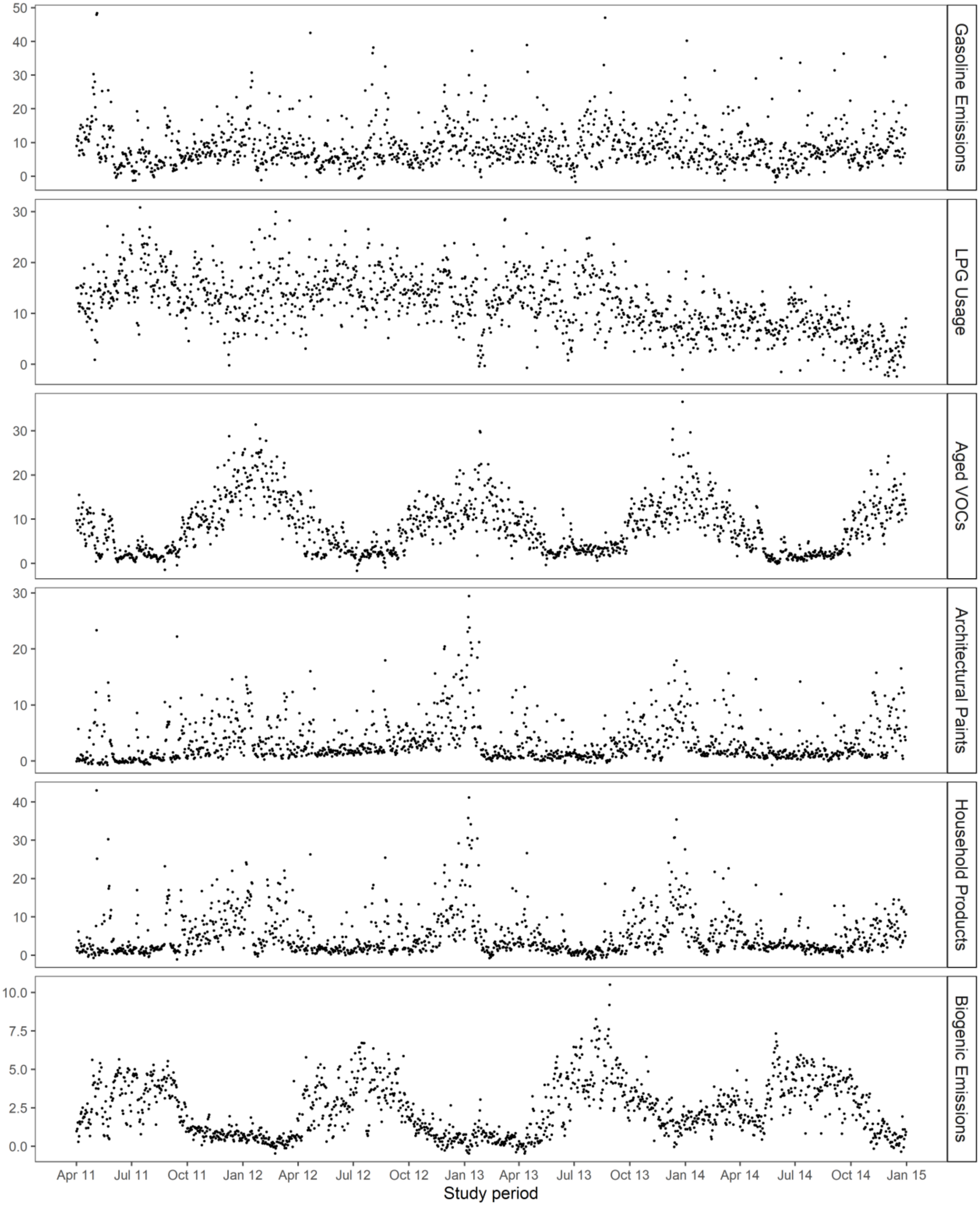

Fig. S2: Time series plots of the daily mean concentrations of VOC sources in Hong Kong, 2011-2014.

4. Sensitivity analyses for the associations

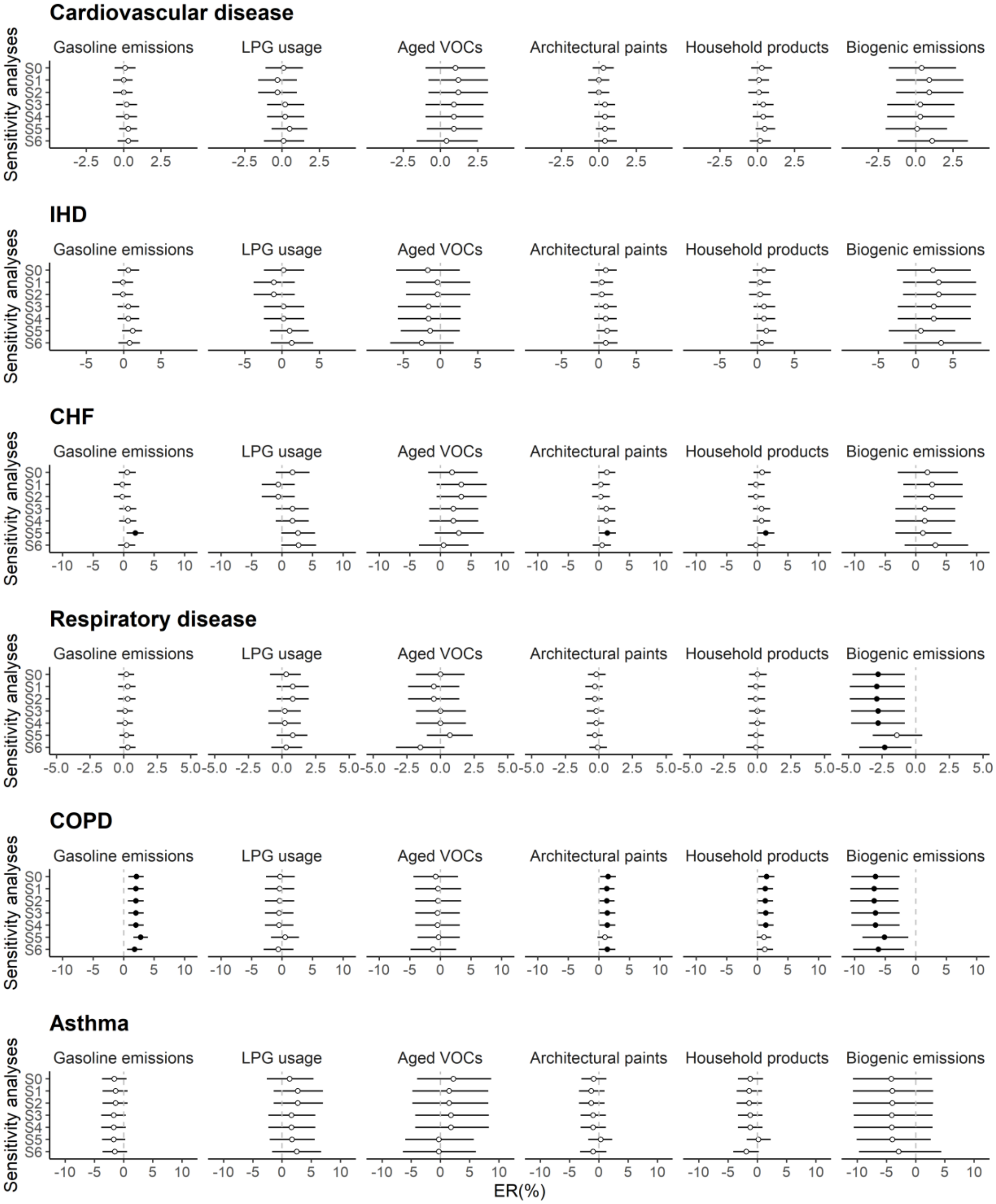

Fig. S3: Sensitivity analyses for the associations of VOC sources with emergency hospital admissions for total and cause-specific diseases in Hong Kong, 2011-2014. S0 is main results in distributed-lag 0-2 days (lag_{0-2}). S1 and S2 are models adjusting for one-week and two-week lags of temperature, respectively. S3 and S4 are models controlling for one-week and two-week lags of ozone. S5 and S6 are models with four and twelve degrees of freedom to filter out secular and seasonal trends, respectively.

5. Age and sex stratified analyses

Table S1. Percent excess risks (%) of emergency hospital admissions for cardiovascular and respiratory diseases with VOC sources at distributed 0-2 lags across seasons in Hong Kong from 2011 to 2014 [a].

	Age subgroups			Sex subgroups		
	Age < 65	Age ≥ 65	P-value [b]	Female	Male	P-value [b]
Cardiovascular disease						
Gasoline emissions	-0.1 (-1.3, 1.0)	0.3 (-0.5, 1.0)	0.562	-0.1 (-1.0, 0.8)	0.4 (-0.5, 1.2)	0.483
LPG usage	-0.9 (-3.0, 1.3)	0.5 (-0.9, 2.0)	0.294	0 (-1.7, 1.8)	0.1 (-1.5, 1.7)	0.984
Aged VOCs	-1.3 (-4.7, 2.2)	1.7 (-0.6, 4.0)	0.161	1.1 (-1.6, 3.9)	-0.3 (-2.8, 2.3)	0.471
Architecture paints	0.1 (-1.1, 1.3)	0.5 (-0.3, 1.3)	0.602	0.4 (-0.6, 1.4)	0.4 (-0.5, 1.3)	0.975
Household products	0 (-1.2, 1.2)	0.5 (-0.3, 1.3)	0.509	0.5 (-0.4, 1.5)	0.2 (-0.7, 1.1)	0.636
Biogenic emissions	4.1 (0.2, 8.1)	-0.9 (-3.4, 1.6)	0.033	0.8 (-2.2, 4.0)	1.3 (-1.6, 4.2)	0.847
IHD						
Gasoline emissions	-0.5 (-3.1, 2.1)	1.1 (-0.6, 2.8)	0.318	0 (-2.2, 2.3)	1.0 (-0.8, 2.8)	0.525
LPG usage	-0.8 (-5.7, 4.2)	0.6 (-2.5, 3.9)	0.625	1.1 (-3.2, 5.5)	-0.4 (-3.7, 3.0)	0.610
Aged VOCs	-5.4 (-12.8, 2.6)	-0.4 (-5.3, 4.7)	0.291	-2.9 (-9.5, 4.1)	-2.3 (-7.4, 3.2)	0.879
Architecture paints	0.9 (-1.9, 3.7)	1.0 (-0.8, 2.7)	0.949	0.6 (-1.8, 3.1)	1.1 (-0.8, 3.1)	0.743
Household products	0.4 (-2.3, 3.2)	1.1 (-0.6, 2.8)	0.688	0.8 (-1.6, 3.2)	1.1 (-0.7, 3.0)	0.838
Biogenic emissions	8.1 (-0.9, 17.8)	0.1 (-5.4, 6.0)	0.147	-0.1 (-7.5, 7.9)	4.9 (-1.2, 11.4)	0.323
CHF						
Gasoline emissions	0.4 (-3.4, 4.4)	0.6 (-0.9, 2.1)	0.920	-0.2 (-2.0, 1.7)	0.8 (-1.2, 2.9)	0.483
LPG usage	0.3 (-6.9, 8.0)	1.9 (-1.0, 4.8)	0.695	2.0 (-1.5, 5.6)	2.6 (-1.3, 6.7)	0.836
Aged VOCs	-0.1 (-10.8, 11.8)	2.2 (-2.0, 6.6)	0.713	0.6 (-4.5, 6.1)	-0.2 (-5.9, 5.8)	0.838
Architecture paints	0.1 (-3.9, 4.1)	1.4 (-0.1, 3.0)	0.534	0.2 (-1.7, 2.2)	1.3 (-0.8, 3.5)	0.444
Household products	0.1 (-3.8, 4.1)	0.8 (-0.7, 2.3)	0.742	-0.2 (-2.1, 1.7)	1.2 (-0.9, 3.3)	0.314
Biogenic emissions	9.1 (-4.1, 24.2)	0.9 (-4.2, 6.3)	0.270	2.5 (-3.7, 9.2)	2.8 (-4.2, 10.3)	0.963
Respiratory disease						
Gasoline emissions	0.1 (-0.9, 1.1)	0.3 (-0.5, 1.0)	0.764	0.3 (-0.5, 1.2)	0.4 (-0.4, 1.1)	0.930
LPG usage	0.8 (-1.1, 2.7)	0.2 (-1.2, 1.6)	0.616	0.3 (-1.3, 1.9)	0 (-1.4, 1.5)	0.840
Aged VOCs	-0.6 (-3.5, 2.4)	-0.1 (-2.3, 2.1)	0.805	-3.0 (-5.5, -0.4)	-1.4 (-3.6, 0.9)	0.360
Architecture paints	-0.1 (-1.2, 1.0)	-0.3 (-1.1, 0.5)	0.796	-0.5 (-1.4, 0.5)	-0.1 (-0.9, 0.7)	0.538
Household products	0.2 (-0.9, 1.2)	-0.1 (-0.9, 0.6)	0.636	-0.4 (-1.3, 0.5)	0.1 (-0.7, 0.9)	0.396
Biogenic emissions	-4.5 (-7.5, -1.3)	-2.4 (-4.8, 0.1)	0.305	-1.7 (-4.4, 1.1)	-3.4 (-5.8, -1.0)	0.360
COPD						
Gasoline emissions	0.8 (-2.7, 4.5)	2.2 (0.9, 3.5)	0.487	2.9 (0, 5.9)	2.0 (0.6, 3.4)	0.568
LPG usage	-1.4 (-7.9, 5.6)	-0.2 (-2.7, 2.3)	0.752	-2.6 (-7.8, 2.9)	-0.3 (-2.9, 2.3)	0.461
Aged VOCs	-6.4 (-16, 4.4)	-0.2 (-4, 3.7)	0.281	3.1 (-5.5, 12.4)	-2.7 (-6.6, 1.4)	0.243
Architecture paints	3.1 (-0.7, 7.0)	1.3 (0, 2.7)	0.405	2.5 (-0.6, 5.7)	1.3 (-0.1, 2.8)	0.512
Household products	3.1 (-0.6, 7.0)	1.3 (0, 2.6)	0.369	1.7 (-1.3, 4.7)	1.4 (0, 2.9)	0.875
Biogenic emissions	-11.6 (-21.5, -0.4)	-6.0 (-10.1, -1.8)	0.347	-7.0 (-15.8, 2.8)	-6.5 (-10.7, -2.0)	0.921
Asthma						
Gasoline emissions	-0.2 (-2.8, 2.5)	-3.7 (-7.0, -0.4)	0.107	-2.7 (-5.4, 0)	0 (-3.0, 3.2)	0.177
LPG usage	0.5 (-4.4, 5.6)	3.5 (-3.0, 10.4)	0.477	1.1 (-3.9, 6.5)	0.4 (-5.3, 6.3)	0.842
Aged VOCs	1.6 (-6.0, 9.9)	2.4 (-7.3, 13.0)	0.915	-0.9 (-8.6, 7.5)	-3.8 (-12.3, 5.5)	0.631
Architecture paints	0.2 (-2.4, 3.0)	-3.0 (-6.4, 0.5)	0.149	-3.2 (-6.0, -0.3)	0 (-3.1, 3.3)	0.142
Household products	0.3 (-2.4, 3.0)	-3.6 (-6.9, -0.1)	0.083	-1.9 (-4.6, 0.9)	-1.3 (-4.4, 1.9)	0.774
Biogenic emissions	-3.3 (-11.3, 5.5)	-7.8 (-17.9, 3.5)	0.514	1.6 (-7.2, 11.3)	-7.2 (-16.3, 2.8)	0.193

[a] distributed-lag model over the previous three days (lag0-2).

[b] Wald test, to test the significance of difference across seasons.
